# Supplementary material for: Open-source models for development of data and metadata standards
Source: Patterns (N Y). 2025 Jul 11;6(7):101316. doi: 10.1016/j.patter.2025.101316 (PMC12416081; doi:10.1016/j.patter.2025.101316)
Supplement: Document S1. Table S1 [file mmc1.pdf]

Patterns, Volume *6*

Supplemental information

Open-source models for development of data and metadata standards

Ariel Rokem, Vani Mandava, Nicoleta Cristea, Anshul Tambay, Kristofer Bouchard, Carolina Berys-Gonzalez, and Andy Connolly

Table S1: List of participants

Name	Affiliation
Alex D Wade	Digital Science
Alexander Szalay	Johns Hopkins University
Andrew Connolly	University of Washington
Anshul Tushar Tambay	University of Washington
Ariel Rokem	University of Washington
Carolina Lorena Berys	University of California, San Diego
Christine Kirkpatrick	San Diego Supercomputer Center
Fernando Seabra Chirigati	Nature Computational Science
Jessica Morgan	NOAA
John Relph	NOAA
Julia Ferraioli	Open Source Stories
Jurriaan Hein Spaaks	formerly Netherlands eScience Center
Justin (Jay) Hnilo	Department of Energy
Kalynn Elisabeth Kennon	Infectious Diseases Data Observatory
Kevin Christopher Booth	Radiant Earth
Kristofer E. Bouchard	Lawrence Berkeley National Labs/UC Berkeley
Lea A. Shanley	University of California, Berkeley
Michael Spannowsky	Durham University
Nicoleta C Cristea	University of Washington
Nina Amla	NSF
Oliver Ruebel	Lawrence Berkeley National Labs
Ray E. Habermann	Metadata Game Changers
Raymond (Ray) Plante	NIST
Robert Hanisch	NIST
Saskia de Vries	Allen Institute for Neural Dynamics
Steven Crawford	NASA
Vani Mandava	University of Washington
Yaroslav O. Halchenko	Dartmouth College
Ziheng Sun	George Mason University
