# Supplementary material for: Open-source models for development of data and metadata standards
Source: Patterns (N Y). 2025 Jul 11;6(7):101316. doi: 10.1016/j.patter.2025.101316 (PMC12416081; doi:10.1016/j.patter.2025.101316)
Supplement: Document S2. Article plus supplemental information [file mmc2.pdf]

Patterns

CellPress
OPEN ACCESS

Review

Open-source models for development of data and metadata standards

Ariel Rokem,[1,2] Vani Mandava,[2,3] Nicoleta Cristea,[2,4] Anshul Tambay,[2,3,*] Kristofer Bouchard,[5,6]
Carolina Berys-Gonzalez,[7] and Andy Connolly[8]
[1]University of Washington, Department of Psychology, Seattle, WA, USA
[2]University of Washington, eScience Institute, Seattle, WA, USA
[3]University of Washington, Scientific Software Engineering Center, Seattle, WA, USA
[4]University of Washington, Department of Civil and Environmental Engineering, Seattle, WA, USA
[5]Lawrence Berkeley National Laboratory, Biological Systems & Engineering Division, Berkeley, CA, USA
[6]University of California, Berkeley, Helen Wills Neuroscience Institute & Redwood Center for Theoretical Neuroscience, Berkeley, CA, USA
[7]University of California, San Diego, Scripps Institution of Oceanography, San Diego, CA, USA
[8]University of Washington, Department of Astronomy, Seattle, WA, USA
*Correspondence: anshul37@uw.edu
https://doi.org/10.1016/j.patter.2025.101316

THE BIGGER PICTURE Data standards are the rules and agreements that govern how data from a particular field are stored and described. These often also extend to metadata—"data about data"—which are associated information about the data that tells users how they were collected and how different parts should be interpreted. Standards are increasingly important in an age in which researchers in various fields are interested in accessing large amounts of data and integrating data of different kinds. There are many ways to develop standards for data and metadata. This review focuses on the adoption of approaches and methods that are taken from the field of open-source software (OSS) development. OSS is a dominant software development mode, and many of the world's largest commercial, academic, and governmental computer systems run on OSS. One of the reasons for its success is the technical tools and social frameworks established by the community that allow different stakeholders to contribute collaboratively. Here, we describe some of the ways in which the communities that create and evolve data and metadata standards for different types of data have been inspired by the way OSS evolves. Standards creators have borrowed social and technical arrangements from OSS, such as the use of versioning systems or approaches to community consensus. Based on a workshop that the authors organized in April 2024, we provide a multidisciplinary and multisector perspective on this topic. We survey instances of such crossover in several different domains: neuroscience, astronomy, high-energy physics, and earth science. We identify challenges for developing data and metadata standards with the OSS model and distill recommendations for various stakeholders about how to leverage OSS approaches toward standards development.

SUMMARY

Machine learning and artificial intelligence promise to accelerate research and understanding across many scientific disciplines. Harnessing the power of these techniques requires aggregating scientific data. In tandem, the importance of open data for reproducibility and scientific transparency is gaining recognition, and data are increasingly available through digital repositories. Leveraging efforts from disparate data collection sources, however, requires interoperable and adaptable standards for data description and storage. Through the synthesis of experiences in astronomy, high-energy physics, earth science, and neuroscience, we contend that the open-source software (OSS) model provides significant benefits for standard creation and adaptation. We highlight resultant issues, such as balancing flexibility vs. stability and utilizing new computing paradigms and technologies, that must be considered from both the user and developer perspectives to ensure pathways for recognition and sustainability. We recommend supporting and recognizing the development and maintenance of OSS data standards and software consistent with widely adopted scientific tools.

This is an open access article under the CC BY license (http://creativecommons.org/licenses/by/4.0/).

INTRODUCTION

Data-intensive discovery has become an important mode of knowledge production across many research fields and has a significant and broad impact across all of society. This is becoming increasingly salient as recent developments in machine learning and artificial intelligence (AI) promise to increase the value of large, multi-dimensional, heterogeneous data sources. Coupled with new machine learning techniques, these datasets can help us understand everything from the cellular operations of the human body, through business transactions on the internet, to the structure and history of the universe. However, the development of new machine learning methods and data-intensive discovery more generally depends on the findability, accessibility, interoperability, and reusability (FAIR) of data[1] as well as metadata.[2] One of the main mechanisms through which the FAIR principles are promoted is the development of standards for data and metadata. Standards can vary in the level of detail and scope and encompass such things as file formats for the storage of certain data types, schemas for databases that organize data, ontologies to describe and organize metadata in a manner that connects them to field-specific meaning, and mechanisms to describe the provenance of analysis products.

Community-driven development of robust, adaptable, and useful standards draws significant inspiration from the development of open-source software (OSS) and has many parallels and overlaps with OSS development. OSS has a long history going back to the development of the Unix operating system in the late 1960s. Over the decades since its inception, the large community of developers and users of OSS has developed a host of socio-technical mechanisms that support the development and use of OSS. For example, the Open Source Initiative (OSI), a non-profit organization that was founded in the 1990s, developed a set of guidelines for the licensing of OSS that is designed to protect the rights of developers and users. On the technical side, tools such as the Git source-code management system support complex and distributed open-source workflows that accelerate and streamline OSS development and make it more robust. Governance approaches have been honed to address the challenges of managing a range of stakeholder interests and mediate between large numbers of weakly connected individuals that contribute to OSS. When these social and technical innovations are put together, they enable a host of positive defining features of OSS, such as transparency, collaboration, and decentralization. These features allow OSS to have a remarkable level of dynamism and productivity while also retaining the ability of a variety of stakeholders to guide the evolution of the software to take their needs and interests into account.

To explore the potential intersection of OSS tools and practices with data and metadata standards, we convened a workshop at the US National Science Foundation (NSF) headquarters on April 8th and 9th, 2024 (with funding through NSF grant #2334483 from the NSF Pathways to Enable Open-Source Ecosystems [POSE] program). Attendees from a broad range of research disciplines and from various sectors (e.g., academic, government, and industry) participated in the meeting (see Table S1 for the list of participants and their affiliations), which included a mix of activities: plenary talks from experts in the field who shared their experience developing open-source data and metadata standards, breakout discussions on a variety of subtopics (e.g., governance of data and metadata standards, strategies for evolving and adapting data and metadata standards, etc.), brainstorming sessions, and networking sessions. The present article is based on a synthesis of notes taken during this workshop and subsequent work by the authors.

We conclude that data and metadata standards that use tools and practices of OSS (“open-source standards” henceforth) reap many of the benefits that the OSS model has provided in the development of other technologies. Here, we explore how OSS processes and tools have affected the development of data and metadata standards in the context of four science use cases: astronomy, high-energy physics (HEP), earth science, and neuroscience. This is based, among other considerations, on the expertise of the authors of this paper, some of whom have participated in the development of open-source standards. For example, neuroscience is heavily represented, as A.R. has participated in the development of the brain imaging data structure (BIDS) standard,[3] and K.B. has been instrumental in the development of the Neurodata Without Borders standard.[4] All authors are heavily involved in OSS development. In particular, V.M. and A.T. are both members of the staff of the recently established Scientific Software Engineering Center at UW (where V.M. is the head of engineering and A.T. is a technical program manager). Other perspectives represented here are via the expertise of the authors in the technical areas of astronomy (A.C.) and earth sciences (N.C. and C.B.-G.).

From our examination of open-source standards, we identify both opportunities and potential challenges in OSS. Issues include the flexibility vs. stability of software, incorporation and utilization of data instrumentation, cultural and technical mismatches between standards developers and user communities, and unclear pathways for standards success and sustainability. We acknowledge that the survey we provide may include some gaps and blind spots, particularly in fields where the authors are less versed (e.g., with respect to the complex array of standards for electronic healthcare records). Nevertheless, to address the issues that we have identified, we distill several recommendations. Specifically, we recommend establishing standards governance based on OSS best practices, fostering meta-standards development, and developing standards in tandem with standards-associated software. On the policy side, we recommend funding the development of open-source standards as well as investment in data stewards and managing cross-sector alliances. Together, these recommendations will ensure that open-source standards development thrives and reaches its full potential.

USE CASES

To understand how OSS development practices affect the development of data and metadata standards, it is informative to demonstrate this cross-fertilization through a few use cases. As we will see in these examples, some fields, such as astronomy, HEP, and earth sciences, have a relatively long history of shared data resources from organizations such as the Sloan Digital Sky Survey (SDSS), Conseil Européen pour la Recherche

CellPress
OPEN ACCESS

Nucléaire (CERN), and National Aeronautics and Space Administration (NASA), while other fields have only relatively recently become aware of the value of data sharing and its impact. These disparate histories inform how standards have evolved and how OSS practices have pervaded their development. They also demonstrate field-specific limitations on the adoption of OSS tools and practices that exemplify some of the challenges, which we will subsequently explore.

Astronomy

An early prominent example of a community-driven standard is the FITS (flexible image transport system) file format standard, which was developed in the late 1970s and early 1980s[5] and has been adopted worldwide for astronomy data preservation and exchange. Essentially every software platform used in astronomy reads and writes the FITS format. It was developed by observatories in the 1980s to store image data in the visible and X-ray spectrum. It has been endorsed by the International Astronomical Union (IAU), as well as funding agencies. Though the format has evolved over time, the commonly used adage "once FITS, always FITS" reveals that the format cannot be evolved to introduce changes that break backward compatibility. Among the features that make FITS so durable is that it was designed originally to have a very restricted metadata schema. FITS records were designed to accommodate the lowest common denominator of word lengths in computer systems at the time. Though compact, FITS files can encode a coordinate frame for pixels, meaning that data from different observational instruments can be stored in this format, and the relationships between the data can be defined, rendering manual and error-prone procedures for conforming images obsolete. This stability has also raised some issues as the field continues to adapt to new measurement methods and the demands of ever-increasing data volumes and complex data analysis use cases, such as interchange with other data and the use of complex databases to store and share data.[6]

Another prominent example of the use of open-source processes to develop standards in astronomy is in the tools and protocols developed by the International Virtual Observatory Alliance (IVOA; https://webtest.ivoa.info/) and its national implementations, e.g., in the US Virtual Astronomical Observatory.[7] The virtual observatories facilitate discovery and access across observatories around the world and underpin data discovery in astronomy. The IVOA took inspiration from the World Wide Web Consortium (W3C; https://www.w3.org/) and adopted its process for the development of its standards (i.e., working drafts, proposed recommendations, and recommendations), with individual standards developed by inter-institutional and international working groups. One of the outcomes of the coordination effort is the development of an ecosystem of software tools developed within both the observatory teams and the user community that interoperate with the standards that were adopted by the observatories.

HEP

Because data collection is centralized, standards to collect and store HEP data have been established, and the adoption of these standards in data analysis has high penetration.[8] A top-down approach is taken so that within every large collaboration, standards are enforced, and this adoption is centrally managed. Access to raw data is essentially impossible because of their large volume, and making them publicly available would be technically very difficult. Therefore, analysis tools are tuned specifically to the standards of the released data. Incentives to use the standards are provided by funders that require data management plans that specify how the data are shared (i.e., in a standards-compliant manner).

Earth sciences

The need for geospatial data exchange between different systems began to be recognized in the 1970s and 1980s, but proprietary formats still dominated. Coordinated standardization efforts brought about the establishment of the Open Geospatial Consortium (OGC; https://www.ogc.org/) in the 1990s, a critical step toward open standards for geospatial data. The 1990s have also seen the development of key standards, such as the network common data form (NetCDF; https://www.unidata.ucar.edu/software/netcdf/) developed by the University Corporation for Atmospheric Research (UCAR; https://www.ucar.edu/) and the hierarchical data format (HDF; https://www.hdfgroup.org/), a set of file formats (HDF4 and HDF5) that are widely used, particularly in climate research. The GeoTIFF format, which originated at NASA in the late 1990s, is extensively used to share image data. The following two decades, the 2000s–2020s, brought an expansion of open standards and integration with web technologies developed by OGC, as well as other standards such as the keyhole markup language (KML) for displaying geographic data in Earth browsers. Formats suitable for cloud computing also emerged, such as the Cloud Optimized GeoTIFF (COG), followed by Zarr and Apache Parquet for array and tabular data, respectively. In 2006, the Open Source Geospatial Foundation (OSGeo, https://www.osgeo.org) was established, demonstrating the community's commitment to the development of open-source geospatial technologies. While some standards have been developed in the industry (e.g., KML by Keyhole, which Google later acquired), they later became international standards of the OGC, which now encompasses more than 450 commercial, governmental, non-profit, and research organizations working together on the development and implementation of open standards.

Neuroscience

In contrast to the previously mentioned fields, neuroscience has traditionally been a "cottage industry," where individual labs have generated experimental data designed to answer specific experimental questions. While this model still exists, the field has also seen the emergence of new modes of data production that focus on generating large, shared datasets designed to answer many different questions, more akin to the data generated in large astronomy data collection efforts.[9] This change has been brought about through a combination of technical advances in data acquisition techniques, which now generate large and very high-dimensional/information-rich datasets; cultural changes that have ushered in new norms of transparency and reproducibility; and funding

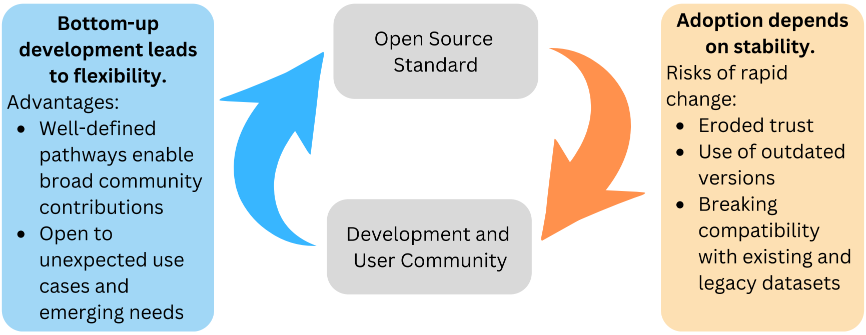

Figure 1. Balancing flexibility and stability in open-source standards

initiatives that have encouraged this kind of data collection. As these changes are recent relative to the other cases mentioned above, standards for data and metadata in neuroscience have been prone to adopting many elements of modern OSS development. Two salient examples in neuroscience are the Neurodata Without Borders file format for neurophysiology data[4] and the BIDS standard for neuroimaging data.[3] BIDS owes some of its success to the adoption of OSS development mechanisms.[10] For example, small changes to the standard are managed through the GitHub pull request mechanism, and larger changes are managed through a BIDS enhancement proposal (BEP) process that is directly inspired by the Python programming language community's Python EP procedure (used to introduce new ideas into the language). Though the BEP mechanism takes a slightly different technical approach, it tries to emulate the open-ended and community-driven aspects of Python development to accept contributions from a wide range of stakeholders and tap a broad base of expertise.

Community science

Another interesting use case for open-source standards is community/citizen science. An early example of this approach is OpenStreetMap (https://www.openstreetmap.org), which allows users to contribute to project development with code and data and to freely use the maps and other related geospatial datasets. But this example is not unique. Overall, this approach has grown in the last 20 years and has been adopted in many different fields. It has many benefits for both the research field, harnessing the energy of non-scientist members of the community to engage with scientific data, and the community members themselves, who can draw both knowledge and pride from their participation in the scientific endeavor. It follows that unique, broader benefits are accrued from this mode of scientific research through the inclusion of perspectives and data that would not otherwise be included. To make data accessible to community scientists and to make the data collected by community scientists accessible to professional scientists, they need to be provided in a manner that can be created and accessed without specialized instruments or specialized knowledge. Here, standards are needed to facilitate interactions between an in-group of expert researchers who generate and curate data and a broader set of out-group enthusiasts who would make meaningful contributions to the science. This creates a particularly stringent constraint on transparency and simplicity of standards. Creating these standards in a manner that addresses these unique constraints can benefit from OSS tools, with the caveat that some of these tools require additional expertise. For example, if the standard is developed using git/GitHub for versioning, this would require learning the complex and obscure technical aspects of these systems that are far from easy to adopt, even for many professional scientists.

OPPORTUNITIES AND RISKS FOR OPEN-SOURCE STANDARDS

While open-source standards benefit from the technical and social aspects of OSS, these tools and practices are associated with risks that need to be mitigated.

Flexibility vs. stability

One of the defining characteristics of OSS is its dynamism and rapid evolution. Because OSS can be used by anyone and, in most cases, contributions can be made by anyone, innovations flow into OSS in a bottom-up fashion from users/developers (Figure 1). Pathways to contribution by members of the community are often well defined from the technical perspective (e.g., through a pull request on GitHub or other similar mechanisms), social perspective (e.g., whether contributors need to accept certain licensing conditions through a contributor licensing agreement), and socio-technical perspective (e.g., how many people need to review a contribution, what are the timelines for a contribution to be reviewed and accepted, what are the release cycles of the software that make the contribution available to a broader community of users, etc.). Similarly, open-source standards may also find themselves addressing use cases and solutions that were not originally envisioned through bottom-up contributions of members of a research community to which the standard pertains. While this dynamism provides an avenue for flexibility, it also presents a source of tension. This is because data and metadata standards apply to already existing datasets, and changes may affect the compliance of these existing datasets. These existing datasets may have a lifespan of decades, making continued compatibility crucial. Similarly, analysis technology stacks that are developed based on an existing version of a standard must adapt to the introduction of new ideas and changes into a standard. Dynamic changes of this sort therefore risk causing a loss of faith in the standard by a user community and migration away from the standard. Additionally, if a standard evolves too rapidly, users may choose to stick to an outdated version of a standard for a long time, creating strains on the community of developers and maintainers of a standard who will need to accommodate long deprecation cycles. In cases where some forms of dynamic change are prohibited—as in the case of the FITS file format, which prohibits changes that break

CellPress
OPEN ACCESS

Table 1. Comparison between standards developers and user communities

	Standards developers	User communities
Technical knowledge	high technical expertise	less or no technical expertise
Motivation	optimize for development and minimize maintenance burden	align with domain-specific requirements and practical applications
Mismatch challenges	may overlook user community needs	potential exclusion from feedback and participation

backward compatibility—there are also costs associated with stability,[6] such as limiting the adoption and combinations of new types of measurements, new analysis methods, or new modes of data storage and data sharing.

Mismatches between standards developers and user communities

Open-source standards often entail an inherent gap between the core developers of the standard and the users of the standard (Table 1). The former may possess a higher ability to engage with the technical details undergirding standards and their development, while the latter still has a high level of interest as members of the broader research field to which the standard pertains. This gap creates friction on the path to broad adoption and best utilization of the standards. In extreme cases, the interests of researchers and standards developers may even seem at odds, as developers implement sophisticated mechanisms to automate the creation and validation of the standard or advocate for more technically advanced mechanisms to evolve the standard. These advanced capabilities offer more robust development practices and consistency in cases where the standards are complex and elaborate. They can also ease the maintenance burden of the standard. On the other hand, they may end up leaving potential experimental researchers and data providers sidelined in the development of the standard and limit their ability to provide feedback about the practical implications of changes to the standards. One example of this (already mentioned above in the use cases section) is the use of git/GitHub for versioning of standards documents. This sets a high bar for participation in standards development for researchers in fields of research in which git/GitHub have not yet had significant adoption as tools of day-to-day computational practice. Another layer of potential mismatches arises when a more complex set of stakeholders needs to be considered. For example, the Group on Earth Observations (GEO; https://earthobservations.org/) is a network that aims to coordinate decision-making around satellite missions and standardize the data that result from these missions. Because this group involves a range of different stakeholders, including individuals who more closely understand potential legal issues and researchers who are better equipped to evaluate technical and domain questions, communication is slow and hindered. As the group aims to move forward by consensus, these communication difficulties can slow down progress. This exemplifies the many cases in which OSS processes that strive for consensus can slow progress.

Cross-domain gaps

There is much to be gained from the development of standards that apply in multiple different domains. For example, many research fields use images as data, and array-based computing that is applicable to images in various research fields is at the core of many scientific codebases. Practitioners within any given field should be motivated to draw on shared data standards and shared software implementations of operations that are common across fields. However, it is hard to justify the investment in these common resources. In principle, data standardization investment is even more justified if the standard is generalizable beyond any specific science domain. In practice, while the use cases are domain-sciences based, data standardization is seen as a data infrastructure and not a science investment, reducing the immediate incentives for researchers to engage with such efforts. This is exacerbated by science research funding schemes that eschew generalized cross-domain solutions and seek to generate tangible impact only with a specific domain.

Data instrumentation issues

Where there is commercial interest in the development of data analysis tools (e.g., in biomedical applications or applications where research funding can be directed toward commercial solutions), there is an incentive to create data formats and data analysis platforms that are proprietary. This may drive innovative applications of scientific measurements but also creates sub-fields where scientific observations are generated by proprietary instrumentation due to these commercialization or other profit-driven incentives. Fourier transform infrared spectroscopy (FTIR spectroscopy) is one such example, wherein the use of Bruker instrumentation necessitates downstream analysis of the resulting measurements using proprietary binary formats necessary for the OPUS Software. Another example is the proliferation of proprietary file formats in electrophysiological measurements of brain signals.[11] Yet another one is the proprietary application programming interfaces (APIs) used in electronic health records.[12] In most cases, there is a lack of regulatory oversight to adhere to available standards or evolve common tools, limiting integration across different measurements. In cases where a significant amount of data is already stored in proprietary formats or where access is limited by proprietary APIs, significant data transformations may be required to get data to a state that is amenable to open-source standards. In these sub-fields, there may also be a lack of incentive to set aside resources to invest in establishing open-source data standards, leaving these sub-fields relatively siloed.

Harnessing new computing paradigms and technologies

Open-source standards development faces the challenges of adapting to new computing paradigms and technologies. Cloud computing provides a particularly stark set of opportunities and challenges. On the one hand, cloud computing offers practical solutions for many challenges of contemporary data-driven research. For example, the scalability of cloud resources addresses some of the challenges of the scale of data that are produced by

instruments in many fields. The cloud also makes data access relatively straightforward due to the ability to determine data access permissions in a granular fashion. On the other hand, cloud computing requires reinstrumenting many data formats. This is because cloud data access patterns are fundamentally different from the ones that are used in local POSIX-style file systems.

Suspicion of cloud computing comes in two different flavors: the first is by researchers and administrators who may be wary of costs associated with cloud computing and especially with the difficulty of predicting these costs. This can particularly affect scenarios where long-term preservation is required. Projects such as NSF's Cloud Bank seek to mitigate some of these concerns by providing an additional layer of transparency into cloud costs.[13] The other type of objection relates to the fact that cloud computing services, by their very nature, are closed ecosystems that resist portability and interoperability. Some aspects of the services will always remain hidden and available only to the cloud computing service provider. In this respect, cloud computing runs afoul of some of the appealing aspects of OSS. That said, the development of "cloud-native" standards can provide significant benefits in terms of the research that can be conducted. For example, NOAA plans to use cloud computing for integration across the multiple disparate datasets that it collects to build knowledge graphs that can be queried by researchers to answer questions that can only be answered through this integration. Putting all the data "in one place" should help with that.

Adaptation to the cloud in terms of data standards has driven the development of new file formats. A salient example is the ZARR format,[14] which supports random access into array-based datasets stored in cloud object storage, facilitating scalable and parallelized computing on these data. Data standards such as Neurodata Without Borders (NWB) and Open Microscopy Environment (OME) now use ZARR as a backend for cloud-based storage. Other file formats that were once not straightforward to use in the cloud, such as HDF5 and TIFF. have been adapted for cloud use (e.g., through the COG format).

Unclear pathways for standards success and sustainability

The development of open-source standards faces similar sustainability challenges to those faced by OSS that is developed for research. Standards typically develop organically through sustained and persistent efforts from dedicated groups of data practitioners. These include scientists and the broader ecosystem of data curators and users. However, there is no playbook on the structure and components of a data standard or the pathway that moves the implementation of a specific data architecture (e.g., a particular file format) to become a data standard. As a result, data standardization lacks formal avenues for success and recognition, for example, through dedicated research grants (see the recommendations for open-source data and metadata standards section below). This hampers the long-term trajectory that is needed to inculcate a standard into the day-to-day practice of researchers.

CROSS-SECTOR INTERACTIONS

The importance of standards stems not only from discussions within research fields about how research can best be conducted to take advantage of existing and growing datasets but also arises from interactions with stakeholders in other sectors. Several different kinds of cross-sector interactions can be defined as having an important impact on the development of open-source standards.

Governmental policy setting

The development of open practices in research has entailed an ongoing interaction and dialogue with various governmental bodies that set policies for research. For example, for research that is funded by the public, this entails an ongoing series of policy discussions that address the interactions between research communities and the general public. One way in which this manifests in the US specifically is in memos issued by the directors of the White House Office of Science and Technology Policy (OSTP), James Holdren (in 2013)[15] and Alondra Nelson (in 2022).[16] While these memos focused primarily on making peer-reviewed publications funded by the US federal government available to the general public, they also lay an increasingly detailed path toward the publication and general availability of the data that are collected in research that is funded by the US government. The general guidance and overall spirit of these memos dovetail with more specific policy guidance related to data and metadata standards. For example, the importance of standards was underscored in a recent report by the Subcommittee on Open Science of the National Science and Technology Council on the "[d]esirable characteristics of data repositories for federally funded research."[17] The report explicitly called out the importance of "allow[ing] datasets and metadata to be accessed, downloaded, or exported from the repository in widely used, preferably non-proprietary, formats consistent with standards used in the disciplines the repository serves." This highlights the need for data and metadata standards across a variety of different kinds of data. In addition, a report from the National Institute of Standards and Technology on "U.S. Leadership in AI: A Plan for Federal Engagement in Developing Technical Standards and Related Tools" emphasized that—specifically for the case of AI—"U.S. government agencies should prioritize AI standards efforts that are […] Consensus-based, […] Inclusive and accessible, […] Multi-path, […] Open and transparent, […] and [that] result in globally relevant and non-discriminatory standards[…]."[18] The converging characteristics of standards that arise from these reports suggest that considerable thought needs to be given to how standards arise so that these goals are achieved. Importantly, open-source standards seem to well match at least some of these characteristics.

The other side of policies is the implementation of these policies in practice by developers of open-source standards and by the communities to which the standards pertain. A compelling road map toward implementation and adoption of open science practices in general and open-source standards in particular is offered in a blog post authored by the Center for Open Science's co-founder and executive director, Brian Nosek, entitled "Strategy for Culture Change."[19] The core idea is that affecting a turn toward open science requires an alignment of not only incentives and values but also technical infrastructure and user experience. A socio-technical bridge between these pieces, which makes the adoption of standards possible and maybe even easy, and the policy goals arises from a community of practice that

CellPress
OPEN ACCESS

Table 2. Recommendations for open-source data and metadata standards

Audience	Recommendations
Science and technology communities	• establish standards governance based on OSS best practices<br>• foster meta-standards development<br>• develop standards in tandem with standards-associated software
Policymaking and funding entities	• fund the development of open-source standards<br>• invest in data stewards<br>• review open-source standards pathways<br>• manage cross-sector alliances

makes the adoption of standards normative. Once all these pieces are in place, making the adoption of open science standards required through policy becomes more straightforward and less onerous.

Funding

Government-set policy intersects with funding considerations. This is because it is primarily directed toward research that is funded through governmental funding agencies. For example, based on high-level policy guidance, calls for federally funded research often ask for data management plans. Over time, these have become increasingly more detailed, and, for example, NSF- and National Institutes of Health (NIH)-funded researchers are now required to both formulate their plans with more clarity and increasingly also to share data using specified standards as a condition for funding.

There are other ways in which funding relates to the development of open-source standards. For example, through the BRAIN Initiative, the NIH has provided key funding for the development of the BIDS standard in neuroscience. Where large governmental funding agencies may not have the resources or agility required to fund nascent or unconventional ways of formulating standards, funding by non-governmental philanthropies and other organizations can provide alternatives. One example (out of many) is the Chan-Zuckerberg Initiative Program for Essential Open Source Software, which funds foundational OSS projects that have an application in biomedical sciences. Distinct from NIH funding, some of this investment focuses on the development of OSS practices, such as funding for the Arrow Project, which focuses on developing OSS maintenance skills and practices rather than a specific biomedical application.

Industry

Interactions of data and metadata standards with commercial interests may provide specific sources of friction. This is because proprietary/closed formats of data can create difficulty at various transition points: from one instrument vendor to another, from data producer to downstream recipient/user, etc. Alternatively, some cross-sector collaborations with commercial entities may pave the way to robust and useful standards. For example, imaging measurements in human subjects (e.g., in brain imaging experiments) significantly interact with standards for medical imaging, chiefly the Digital Imaging and Communications in Medicine (DICOM) standard, which is widely used in a range of medical imaging applications, including in clinical settings.[20] The standard emerged from the demands of clinical practice in the 1980s as digital technologies came into widespread use in medical imaging through the joint work of industry organizations: the American College of Radiology and the National Association of Electronic Manufacturers. One of the defining features of the DICOM standard is that it allows manufacturers of instruments to define "private fields" that are compliant with the standard but which may include idiosyncratically organized data and/or metadata. This provides significant flexibility but can also easily lead to the loss of important information. Overall, the human brain imaging case is exemplary of a case in which industry standards and research standards coexist and need to communicate with each other effectively to advance research use cases while keeping up with the rapid development of the technologies. Healthcare in particular is a domain in which industry pressures apply strongly within a rapidly evolving technological landscape and with open data models and metadata profiles, such as HL7 Fast Healthcare Interoperability Resources (FHIR),[21] Observational Medical Outcomes Partnership (OMOP),[22] and standardization consortium efforts, such as the Clinical Data Interchange Standards Consortium (CDISC).[23] However, we will not provide a full account of healthcare standards in this context and refer interested readers instead to a survey of this literature.[24] Funding mandates and incentives may play an important role in the industry case as well, as commercial software vendors could become interested in providing solutions that are compliant with funding mandates. Thus, they may be incentivized to align their products with standards, for example, in the manner that samples are described.

RECOMMENDATIONS FOR OPEN-SOURCE DATA AND METADATA STANDARDS

In conclusion, we propose a set of recommendations that distill the lessons learned from the examination of data and metadata standards through the lens of OSS development practices. We divide this section into two parts: one aimed at the science and technology communities that develop and maintain open-source standards and the other aimed at policymaking and funding agencies, who have an interest in fostering more efficient, more robust, and more transparent open-source standards (Table 2).

Science and technology communities

Establish standards governance based on OSS best practices

While best-practice governance principles are relatively new in OSS communities, there is already a substantial set of prior art in this domain on which the developers and maintainers of open-source data and metadata standards can rely. For example, it is now clear that governance principles and rules can mitigate some of the risks and challenges mentioned in the opportunities and risks for open-source standards section above, especially for communities beyond a certain size that need to converge toward a new standard or rely on an existing standard. Developers and maintainers should review existing governance practices, such as those provided by The Open Source Way (https://www.theopensourceway.org/).

Foster meta-standards development

One of the main conclusions from our survey of existing standards is that there is significant knowledge that exists across fields and domains and informs the development of standards within each field but could be surfaced to the level where it may be adopted more widely in different domains and be more broadly useful. One approach to this is a comparative approach: in this approach, a readiness and/or maturity model can be developed that assesses the challenges and opportunities that a specific standard faces at its current phase of development. Developing such a maturity model, while it goes beyond the scope of this paper, could lead to the eventual development of a meta-standard or a standard of standards. This would facilitate a succinct description of cross-cutting best practices that can be used as a basis for the analysis or assessment of an existing standard or as guidelines to develop new standards. For instance, specific barriers to adopting a data standard that considers the size of the community and its specific technological capabilities should be considered.

More generally, meta-standards could include formalization for versioning of standards and interactions with specific related software. This includes amplifying formalization/guidelines on how to create standards (for example, metadata schema specifications using LinkML, https://linkml.io; https://github.com/linkml/). One potential challenge would be to come to agreements about pathways to standardization and formalization across multiple research domains that do not usually communicate with each other. Aspects of communication with potential user audiences (e.g., researchers in particular domains) should be considered as well, such as in the quality of onboarding documentation and tools for ingestion or conversion into standards-compliant datasets even within each domain.

An ontology for the standards development process—e.g., top down vs. bottom up, the minimum number of datasets, target community size, technical expertise typical of this community, and so forth—could help guide the standards development process toward more effective adoption and use. A set of meta-standards and high-level descriptions of the standards development process—some of which are laid out in this paper—could help standard developers avoid known pitfalls, such as the dreaded proliferation of standards or complexity-impeded adoption. Surveying and documenting the successes and failures of current standards for a specific dataset/domain can help disseminate knowledge about the standardization process. Resources such as Fairsharing (https://fairsharing.org/)[25] or the Digital Curation Center (https://www.dcc.ac.uk/guidance/standards) can help guide this process.

Develop standards in tandem with standards-associated software

Development of standards should be coupled and tightly linked with the development of the associated software. This produces a virtuous cycle where the use cases and technical issues that arise in software development inform the development of the standard, and vice versa. One of the lessons learned across a variety of different standards is the importance of automated validation of the standard. Automated validation is broadly seen as a requirement for the adoption of a standard and a factor in managing change of the standard over time. To advance this virtuous cycle, we recommend making data standards machine readable and considering software creation an integral part of establishing a standard's schema. Additionally, standards evolution should maintain software compatibility and the ability to translate and migrate between standards.

Policymaking and funding entities

Fund the development of open-source standards

While some funding agencies already support standards development as part of the development of informatics infrastructures, data standards development should be seen as integral to science innovation and earmarked for funding in research grants, not only in specialized contexts. Funding models should encourage the development and adoption of standards and fund associated community efforts and tools for this. The OSS model is seen as a particularly promising avenue for an investment of resources because it builds on previously developed procedures and technical infrastructure and provides avenues for the democratization of development processes and community input along the way. At the same time, there are significant challenges associated with incentives to engage, ranging from the dilution of credit to individual contributors to the burnout of maintainers and developers. The clarity offered by procedures for EPs and semantic versioning schemes adopted in standards development offers avenues for a range of stakeholders to propose well-defined contributions to large and field-wide standards efforts (e.g., Pestilli et al.[26]) and potentially helps alleviate some of these concerns by providing avenues for individual contributions to surface, as well as for clarity of process, which can alleviate the risks of maintainer burnout.

Invest in data stewards

Advancing the development and adoption of open-source standards requires the dissemination of knowledge to researchers in a variety of fields, but this dissemination itself may not be enough without the fostering of specialized expertise. Therefore, it is important to recognize the distinct role that data stewards play in contemporary research. As policy demands for openness become increasingly high, it is crucial to truly support experts whose role will be to develop, maintain, and facilitate the adoption and use of open-source standards. This support needs to manifest in all stages of the career of these individuals: it will be necessary to set up programs for training for data stewards and to invest in the career paths of individuals who receive such training so that this crucial role is encouraged. Initial proposals for the curriculum and scope of the role have already been proposed (e.g., in Mons[27]), but we also identify here a need to connect these individuals directly to the practices that exemplify open-source standards. Thus, it will be important for these individuals to be conversant in the methodology of OSS. This does not mean that they need to become software engineers—though for some of them, there may be some overlap with the role of research software engineers[28]—but rather that they need to become familiar with those parts of the OSS development life cycle that are specifically useful for the development of open-source standards, such as tools for version control, tools for versioning, and tools for creation and validation of compliant data and metadata. Stakeholder organizations should invest in training grants to establish a curriculum for data and metadata standards education.

Ultimately, efficient use of data stewards and their knowledge will have to be applied. It is evident that not every project and

CellPress
OPEN ACCESS

every lab that produces data requires a full-time data steward. Instead, data stewardship could be centralized within organizations such as libraries, data science cores, or software engineering cores of larger research organizations. This would be akin to recent models for research software engineering that are becoming common in many research organizations.[29] Efficiency considerations also suggest that the development of data standards would not have its intended purpose unless funds are also allocated to the implementation of the standard in practice. Mandating standards without appropriate funding for their implementation by data producers and data users could risk hampering science and could lead to researchers doing the bare minimum to make their data "open."

Review open-source standards pathways

Programs that examine retrospective pathways for establishing data standards should be invested in. The publication of life cycles for successful data standards should be encouraged. These life cycles should include the process, creators, affiliations, grants, and adoption journeys of open-source standards. To encourage sustainable development of open-source standards and to build on prior experience, the documentation and dissemination of life cycles should be seen as an integral step of the work of standards creators and granting agencies. In the meantime, it would be good to also retroactively document the life cycle of existing standards that are seen as success stories and foster awareness of these standards. In addition, fostering research projects on the principles that underlie successful open-source standards development will help formulate new standards and iterate on existing ones. In accordance, data management plans should promote the sharing of not only data but also metadata and descriptions of how to use them.

Manage cross-sector alliances

Cross-sector and cross-domain alliances that can impact successful standards development should be encouraged. Robust program management of these alliances to align the pace and create incentives should be invested in (for instance, via open-source program offices at universities or other research organizations). Like program officers at funding agencies, standards evolution needs sustained project management efforts. Multiparty partnerships should include strategic initiatives for standard establishment, such as the Pistoia Alliance (https://www.pistoiaalliance.org/).

ACKNOWLEDGMENTS

This paper was produced following a workshop held at NSF headquarters in Alexandria, VA, on April 8th and 9th, 2024. We thank the speakers and participants of this workshop for the time and thought that they put into the workshop. A list of workshop participants is provided as Table S1. The workshop and this paper were funded through NSF grant #2334483 from the NSF Pathways to Enable Open-Source Ecosystems (POSE) program. The opinions expressed in this paper do not necessarily reflect those of the NSF.

AUTHOR CONTRIBUTIONS

A.R., V.M., N.C., and A.C. acquired funding. All authors participated in the workshop on which this paper was based and took notes during this workshop. A.R. synthesized the notes and wrote the first draft of the paper. C.B.-G. created the figure. All authors provided comments and suggestions that were included in the finalized manuscript.

DECLARATION OF INTERESTS

The authors declare no competing interests.

SUPPLEMENTAL INFORMATION

Supplemental information can be found online at https://doi.org/10.1016/j.patter.2025.101316.

REFERENCES

1. Wilkinson, M.D., Dumontier, M., Aalbersberg, I.J.J., Appleton, G., Axton, M., Baak, A., Blomberg, N., Boiten, J.W., da Silva Santos, L.B., Bourne, P.E., et al. (2016). The FAIR guiding principles for scientific data management and stewardship. Sci. Data *3*, 160018.

2. Musen, M.A. (2022). Without appropriate metadata, data-sharing mandates are pointless. Nature *609*, 222.

3. Gorgolewski, K.J., Auer, T., Calhoun, V.D., Craddock, R.C., Das, S., Duff, E.P., Flandin, G., Ghosh, S.S., Glatard, T., Halchenko, Y.O., et al. (2016). The Brain Imaging Data Structure, a format for organizing and describing outputs of neuroimaging experiments. Sci. Data *3*, 160044.

4. Rübel, O., Tritt, A., Ly, R., Dichter, B.K., Ghosh, S., Niu, L., Baker, P., Soltesz, I., Ng, L., Svoboda, K., et al. (2022). The Neurodata Without Borders ecosystem for neurophysiological data science. eLife *11*, e78362.

5. Wells, D.C., and Greisen, E.W. (1979). FITS - a flexible image transport system. In International Workshop on Image Processing in Astronomy. Proceedings of the 5th. Colloquium on Astrophysics, held in Trieste, Italy, June 4-8, 1979, G. Sedmak, M. Capaccioli, and R.J. Allen, eds. (Trieste, Italy: Osservatorio Astronomico di Trieste), pp. 445–459.

6. Scroggins, M., and Boscoe, B.M. (2020). Once FITS, always FITS? Astronomical infrastructure in transition. IEEE Ann. Hist. Comput. *42*, 42–54.

7. Hanisch, R.J., Berriman, G.B., Lazio, T.J.W., Emery Bunn, S., Evans, J., McGlynn, T.A., and Plante, R. (2015). The virtual astronomical observatory: Re-engineering access to astronomical data. Astron. Comput. *11*, 190–209.

8. Basaglia, T., Bellis, M., Blomer, J., Boyd, J., Bozzi, C., Britzger, D., Campana, S., Cartaro, C., Chen, G., Couturier, B., et al. (2023). Data preservation in high energy physics. Eur. Phys. J. C *83*, 795.

9. Koch, C., and Reid, R.C. (2012). Observatories of the mind. Nature *483*, 397–398.

10. Poldrack, R.A., Markiewicz, C.J., Appelhoff, S., Ashar, Y.K., Auer, T., Baillet, S., Bansal, S., Beltrachini, L., Benar, C.G., Bertazzoli, G., et al. (2024). The past, present, and future of the brain imaging data structure (BIDS). Imaging Neurosci. *2*, 1–19.

11. Hermes, D., and Cimbalnek, J. (2023). How can intracranial EEG data be published in a standardized format? In Studies in Neuroscience, Psychology and Behavioral Economics, N. Axmacher, ed. (Springer International Publishing), pp. 595–604.

12. Adler-Milstein, J., and Pfeifer, E. (2017). Information blocking: Is it occurring and what policy strategies can address it? Milbank Q. *95*, 117–135.

13. Norman, M., Kellen, V., Smallen, S., DeMeulle, B., Strande, S., Lazowska, E., Alterman, N., Fatland, R., Stone, S., Tan, A., et al. (2021). CloudBank: Managed Services to Simplify Cloud Access for Computer Science Research and Education. In PEARC '21: Practice and Experience in Advanced Research Computing 2021: Evolution Across All Dimensions, J. Paris, J. Milhans, B. Hillery, S.B. Geva, P. Schmitz, and R. Sinkovits, eds. (Association for Computing Machinery), pp. 1–4.

14. Miles, A., jakirkham, Bussonnier, M., Moore, J., Orfanos, D.P., Bennett, D., Stansby, D., Hamman, J., Bourbeau, J., Fulton, A., et al. (2024). zarr-developers/zarr-python: v3.0.0-alpha. Zenodo. https://doi.org/10.5281/zenodo.11592827.

15. Holdren, J.P. (2013). Memorandum for the heads of executive departments and agencies: Increasing access to the results of federally funded scientific research (Office of Science and Technology Policy, USA). https://doi.org/10.21949/1528360.

16. Nelson, A. (2022). Memorandum for the heads of executive departments and agencies: Ensuring free, immediate, and equitable access to federally funded research (Office of Science and Technology Policy, USA). https://doi.org/10.21949/1528361.

17. The National Science and Technology Council (2022). Desirable characteristics of data repositories for federally funded research (Executive Office of the President of the United States). https://doi.org/10.5479/10088/113528.

18. National Institute of Standards and Technology. (2019). U.S. LEADERSHIP IN AI: A plan for federal engagement in developing technical standards and related tools. https://www.nist.gov/system/files/documents/2019/08/10/ai_standards_fedengagement_plan_9aug2019.pdf.

19. Nosek, B. (2019). Strategy for Culture Change (Center for Open Science). https://www.cos.io/blog/strategy-for-culture-change.

20. Mustra, M., Delac, K., and Grgic, M. (2008). Overview of the DICOM standard. In 2008 50th International Symposium ELMAR, *1* (IEEE), pp. 39–44.

21. Bender, D., and Sartipi, K. (2013). In HL7 FHIR: An agile and RESTful approach to healthcare information exchange, P.P. Rodrigues, M. Pechenizkiy, J. Gama, R. Cruz Correia, J. Liu, A. Traina, P. Lucas, and P. Soda, eds. (IEEE), pp. 326–331.

22. Reinecke, I., Zoch, M., Reich, C., Sedlmayr, M., and Bathelt, F. (2021). The usage of OHDSI OMOP - a scoping review. Stud. Health Technol. Inform. *283*, 95–103.

23. Facile, R., Muhlbradt, E.E., Gong, M., Li, Q., Popat, V., Pétavy, F., Cornet, R., Ruan, Y., Koide, D., Saito, T.I., et al. (2022). Use of clinical data interchange standards consortium (CDISC) standards for real world data: Expert perspectives from a qualitative delphi survey. JMIR Med. Inform. *10*, e30363.

24. Schulz, S., Stegwee, R., and Chronaki, C. (2019). Standards in Healthcare Data. In Fundamentals of clinical data science, P. Kubben, M. Dumontier, and A. Dekker, eds. (Springer International Publishing), pp. 19–36.

25. Sansone, S.A., McQuilton, P., Rocca-Serra, P., Gonzalez-Beltran, A., Izzo, M., Lister, A.L., and Thurston, M.; FAIRsharing Community (2019). FAIRsharing as a community approach to standards, repositories and policies. Nat. Biotechnol. *37*, 358–367.

26. Pestilli, F., and Herholz, P. (2024). A community-driven development of the brain imaging data standard (BIDS) to describe macroscopic brain connections. OSF. https://doi.org/10.17605/OSF.IO/U4G5P.

27. Mons, B. (2018). Data Stewardship for Open Science: Implementing FAIR Principles. 1st Edition (CRC Press). https://doi.org/10.1201/9781315380711.

28. Connolly, A., Hellerstein, J., Alterman, N., Beck, D., Fatland, R., Lazowska, E., Mandava, V., and Stone, S. (2023). Software Engineering Practices in Academia: Promoting the 3rs—Readability, Resilience, and Reuse. Harv. Data Sci. Rev. *5*. https://doi.org/10.1162/99608f92.018bf012.

29. Van Tuyl, S. (2023). Hiring, managing, and retaining data scientists and research software engineers in academia: A career guidebook from ADSA and US-RSE. Zenodo. https://doi.org/10.5281/zenodo.8329337.

Patterns, Volume *6*

Supplemental information

Open-source models for development of data and metadata standards

Ariel Rokem, Vani Mandava, Nicoleta Cristea, Anshul Tambay, Kristofer Bouchard, Carolina Berys-Gonzalez, and Andy Connolly

Table S1: List of participants

Name	Affiliation
Alex D Wade	Digital Science
Alexander Szalay	Johns Hopkins University
Andrew Connolly	University of Washington
Anshul Tushar Tambay	University of Washington
Ariel Rokem	University of Washington
Carolina Lorena Berys	University of California, San Diego
Christine Kirkpatrick	San Diego Supercomputer Center
Fernando Seabra Chirigati	Nature Computational Science
Jessica Morgan	NOAA
John Relph	NOAA
Julia Ferraioli	Open Source Stories
Jurriaan Hein Spaaks	formerly Netherlands eScience Center
Justin (Jay) Hnilo	Department of Energy
Kalynn Elisabeth Kennon	Infectious Diseases Data Observatory
Kevin Christopher Booth	Radiant Earth
Kristofer E. Bouchard	Lawrence Berkeley National Labs/UC Berkeley
Lea A. Shanley	University of California, Berkeley
Michael Spannowsky	Durham University
Nicoleta C Cristea	University of Washington
Nina Amla	NSF
Oliver Ruebel	Lawrence Berkeley National Labs
Ray E. Habermann	Metadata Game Changers
Raymond (Ray) Plante	NIST
Robert Hanisch	NIST
Saskia de Vries	Allen Institute for Neural Dynamics
Steven Crawford	NASA
Vani Mandava	University of Washington
Yaroslav O. Halchenko	Dartmouth College
Ziheng Sun	George Mason University
